# Motor cortex stimulation: a systematic literature-based analysis of effectiveness and case series experience

**DOI:** 10.1186/s12883-019-1273-y

**Published:** 2019-03-29

**Authors:** Jia-Jie Mo, Wen-Han Hu, Chao Zhang, Xiu Wang, Chang Liu, Bao-Tian Zhao, Jun-Jian Zhou, Kai Zhang

**Affiliations:** Department of Neurosurgery, Beijing Tiantan Hospital, Capital Medical University, No. 119 South 4th Ring West Road, Fengtai District, Beijing, 100070 China

**Keywords:** Motor cortex stimulation, Neuromodulation, Refractory pain

## Abstract

**Background:**

Aim to quantitatively analyze the clinical effectiveness for motor cortex stimulation (MCS) to refractory pain.

**Methods:**

The literatures were systematically searched in database of Cocharane library, Embase and PubMed, using relevant strategies. Data were extracted from eligible articles and pooled as mean with standard deviation (SD). Comparative analysis was measured by non-parametric t test and linear regression model.

**Results:**

The pooled effect estimate from 12 trials (*n* = 198) elucidated that MCS shown the positive effect on refractory pain, and the total percentage improvement was 35.2% in post-stroke pain and 46.5% in trigeminal neuropathic pain. There is no statistical differences between stroke involved thalamus or non-thalamus. The improvement of plexus avulsion (29.8%) and phantom pain (34.1%) was similar. The highest improvement rate was seen in post-radicular plexopathy (65.1%) and MCS may aggravate the pain induced by spinal cord injury, confirmed by small sample size. Concurrently, Both the duration of disease (*r* = 0.233, *p* = 0.019*) and the time of follow-up (*r* = 0.196, *p* = 0.016*) had small predicative value, while age (*p* = 0.125) had no correlation to post-operative pain relief.

**Conclusions:**

MCS is conducive to the patients with refractory pain. The duration of disease and the time of follow-up can be regarded as predictive factor. Meanwhile, further studies are needed to reveal the mechanism of MCS and to reevaluate the cost-benefit aspect with better-designed clinical trials.

**Electronic supplementary material:**

The online version of this article (10.1186/s12883-019-1273-y) contains supplementary material, which is available to authorized users.

## Background

Refractory pain, resulting from various causes, presents a clinically therapeutic challenge as responding poorly to all types of available pharmacological therapies. With the development neuromodulatory techniques, intracranial and extracranial stimulation were seemed promising. Motor cortex stimulation (MCS) represents an effectively functional neurosurgery to attenuate the various types of neuropathic pain including post-stroke pain, trigeminal neuropathic pain, plexus, phantom pain, pain induced by spinal cord injury and post-radicular plexopathy [1–7]. However, it remains controversial as some published articles shown negative results [6, 8–10]. The practical efficacy different in various centers and the small sample studies together contribute to the uncertain perspective of MCS. To solve the discrepancy, the aim of the present article is to quantitatively evaluate and analyze the clinical effectiveness for MCS to refractory pain.

## Methods

### Literature search

Correlated literatures were systematically retrieved from bibliographic databases, such as Google Scholar, Embase and PubMed, according to the predefined strategies including “motor cortex stimulation” and “pain”. Only literatures descripting the application of MCS in refractory pain were included for further analysis. Furthermore, grey literatures, lecture records and any missed trials were hand-searched.

### Study selection

Only the literatures met the criteria below were retrieved and reviewed for eligibility:

1. Participants: patients diagnosed with refractory pain;
2. Interventions: extradural/subdural motor cortex stimulation (both of them shown the similar effect [11]); without any other surgical treatment;
3. Outcomes: widely accepted and unified evaluation standard-Visual Analog Scale (VAS) was considered as primary outcome measure;
4. Designs: clinical trials with restrict inclusion criteria; small sample studies (*n* < 5) would be removed because it may produce exaggerated intervention effect estimates;
5. Predictive factors: systematical analysis of predictive factors.

### Surgical management

Prior to the MCS operation, all patients underwent skin fiducial marker placement on standard anatomical reference points. The MCS operation was performed under general anesthesia through a small craniotomy over the motor cortex on the side contralateral to the pain. The motor cortex was identified through intraoperative somato-sensory evoked potentials (SSEP). Recording strip were epidurally placed perpendicular to and across the presumed location and direction of the central sulcus. The position was confirmed once the phase reversal was obtained [12]. The stimulation electrode (Medtronic 3587A/39585) was anatomically located in the motor cortical area parallel to the central sulcus. Co-registration of the preoperative and post-operative CT was used to confirmation localization of the electrode using the iElectrodes software (version 1.010) [13]. Eventually, the neurostimulator was permanently implanted subcutaneously in the chest after achieving satisfactory pain relief following temporal stimulation. Approximately 5–7 days after the implantation of the electrode, the stimulator is turned on, and the stimulation parameters depend on the patients’ subjective feelings to maximize the therapeutic effect and avoid side effects.

### Statistical analysis

The data collected from the eligible studies was pooled and analyzed. Post-operative scores would be used to evaluate the efficacy of MCS for pain at short−/long-term period, comparing to the baseline. Changes in VAS scores were summarized for each time period for comparison and presented as mean with standard deviation (SD). To determine if efficacy was significantly different between the different types of neuropathic pain, normality test and homogeneity of variance test would be performed firstly. Then, data accorded with normal distribution and homogeneity of variance were compared by the Student’s t-test, otherwise, by non-parametric t test. Meanwhile, linear regression model would be used to investigate whether the age, duration of disease and time of follow-up could be regarded as predictive factors. All statistical analyses were performed in the Stata 12.0 software (StataCorp LP, College Station, USA), and *p* < 0.05 was considered statistically significant.

## Results

### Search Results

Overall, 2181 literatures initially identified in the databases (up to October 2017) with our search strategy. After independently reading the titles and abstracts of the relevant articles, 172 were included to further investigate. Full text of potentially relevant articles were independently retrieved by each review author, eventually, 12 [10, 11, 14–23] appeared to be eligible to apply MCS in refractory pain. The details of all included studies were demonstrated in Table 1, including the centers, the number of participants, diagnosis, duration of disease and the time of follow- up. Some literatures were excluded as they failed to fulfill our inclusion criteria: 1) without detail information of each patient [1, 3, 4, 24–33]; VAS was not as the primary outcome measures [6, 9, 34–36]; small sample studies (participants less than 5) [37–39]. Moreover, there was the considerable confusion about the efficacy of MCS because four studies [6, 8–10] shown the invalid outcomes. Among included literatures, all reported the age of patients and the preoperative VAS scores [10, 11, 14–23]; seven reported the duration of the disease [11, 16, 17, 19–21, 23]; and 9 reported the time of follow-up [10, 15–17, 19–23]. All the information used in the present study were available in the Additional file 1.

**Table 1:** Summary of literatures results of MCS for refractory Pain

Literatures	Center	Etiology	Duration (months)	Follow-up (months)	Analgesic effect (VAS)	Improvement
Zhang 2017 [1]	Xuanwu Hospital, China	16 Stroke	30.9 ± 28.3	28.2 ± 10.7	8.0 ± 0.7 - > 5.3 ± 2.4	33.67%
Rasche 2016 [2]	University Hospital of Schleswig-Holstein, Germany	36 TNP	NA	91.6 ± 33.6	9.2 ± 0.8 - > 5.0 ± 1.2	45.67%
Sokal 2015 [5]	Military Research Hospital, Poland	6 Stroke; 2 TNP; 3 PA; 1 Phantom pain; 1 MS; 1 Syringomyelia	126.0 ± 82.7	39.4 ± 20.2	8.6 ± 0.6 - > 5.1 ± 2.3	41.03%
Slotty 2015 [6]	Vancouver General Hospital, Canada	11 Stroke; 2 TNP; 4 PA; 4 SCI; 2 CRPS	64.9 ± 45.0	39.2 ± 19.6	7.7 ± 1.3 - > 6.9 ± 2.0	10.58%
Sachs 2014 [17]	The Ottawa Hospital, Canada	1 Stroke; 7 TNP; 3 Phantom pain; 1 Facial hemangiopericytoma; 2 Vascular malformations	NA	12.2 ± 12.4	7.2 ± 1.3 - > 5.7 ± 1.9	18.62%
Delavallee 2014 [19]	Cliniques Universitaires Saint-Luc, Belgium	3 Stroke; 7 TNP; 3 PA; 1 Phantom pain; 3 Trauma; 1 CRPS	153.0 ± 138.6	103.1 ± 44.6	8.8 ± 0.7 - > 1.6 ± 1.2	81.70%
Buchanan 2014 [20]	University Medical Center at Brackenridge, USA	2 Stroke; 5 TNP; 1 Phantom pain	24	36	9.5 ± 0.8 - > 5.5 ± 2.4	41.28%
Velasco 2008 [21]	Mexico General Hospital, Mexico	1 Stroke; 3 PA; 5 Post-herpetic neuralgia; 1 Hemangiectasia syndrome; 1 Scleroderma	79.8 ± 63.6	12	9.5 ± 0.8 - > 3.6 ± 1.5	70.00%
Delavallee 2008 [11]	Cliniques Universitaires Saint-Luc, Belgium	3 Stroke; 3 TNP; 1 PA; 1 Trauma	177 ± 160.5	NA	8.6 ± 0.5 - > 3.0 ± 2.6	65.63%
Rasche 2006 [22]	University Hospital Heidelberg, Germany	7 Stroke; 9 TNP; 6 Surgical injury	66.7 ± 36.9	120	8.6 ± 0.9 - > 6.7 ± 2.5	23.45%
Pirotte 2005 [23]	Universite Libre de Bruxelles, Belgium	6 Stroke; 4 TNP; 1 PA; 2 Spinal syrinx; 1 MS; 2 Amputation; 1 Surgical injury; 1 Post-radicular plexopathy	NA	29.8 ± 16.9	7.5 ± 0.6 - > 3.7 ± 3.1	50.56%
Brown 2005 [24]	Wayne State University School of Medicine, USA	2 Stroke; 4 TNP; 2 Post-herpetic neuralgia	NA	NA	9.0 ± 1.9 - > 3.9 ± 2.9	58.93%

*CRPS* complex regional pain syndrome, *TNP* trigeminal neuropathic pain, *PA* plexus avulsion, *SCI* spinal cord injuries, *MS* multiple sclerosis, *NA* not available, *VAS* visual analogue scale, The data are recorded as mean ± Standard Deviation (SD)

### Clinical outcomes

**Figure 1:**
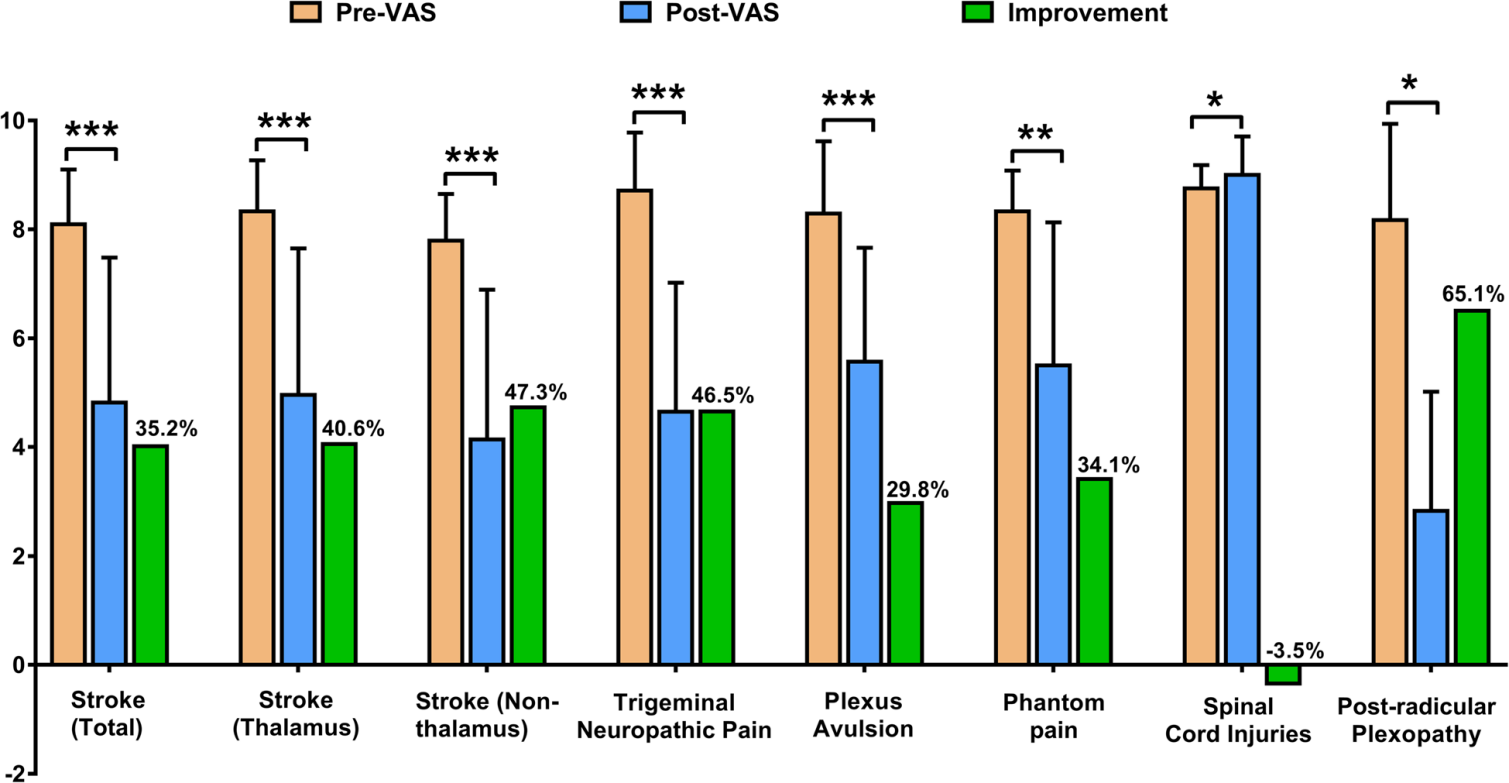
Column graph. Analgesic effect of MCS in different aetiologies of refractory pain. *: *p* < 0.05; **: *p* < 0.01; ***: *p* < 0.001

We considered all patients with refractory pain who underwent MCS and were evaluated with VAS scores. Then, we investigated whether a significant difference existed among the different aetiologies of refractory pain. All subgroup data failed to pass the normality test (the Kolmogorov-Smirnov test or Shapiro-Wilk test depending on the sample size) and the homogeneity test of variance (Levene’s test). A non-parametric test (Wilcoxon signed-rank test) was used to investigate the analgesic effect of MCS. MCS showed a positive effect on refractory pain; the total percentage improvement was 35.2% for post-stroke pain and 46.5% for trigeminal neuropathic pain. For cases of cerebral infarction not located in the thalamus, the mean improvement was 47.3%, which was much higher than that of the cases of cerebral infarction located in the thalamus (40.6%). However, no significant differences were observed between strokes that involved the thalamus or non-thalamus (Kruskal-Wallis test, *p* = 0.700). The improvement of plexus avulsion (29.8%) and phantom pain (34.1%) was similar. The highest improvement rate was seen for post-radicular plexopathy (65.1%). However, MCS may aggravate the pain induced by spinal cord injury (− 3.5%) as confirmed by the small sample size (Fig. 1 & Additional file 2: Figure S1).

**Figure 2:**
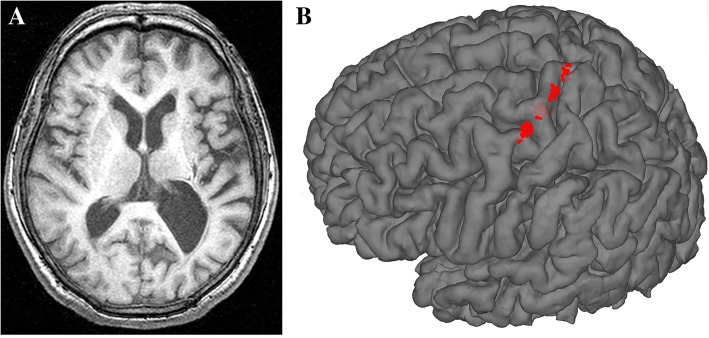
Neuroimaging data from patient 2 (MWH, female, 74 years old, left thalamic hemorrhage 20 years ago); **a** Presurgical T1-weighted MRI (left ventricle enlargement); **b** Three-dimensional reconstruction of electrode placement (left precentral gyrus)

The detailed information of three patients with refractory pain underwent MCS were displayed in Table 2. And the neuroimaging data was shown in Fig. 2.

**Table 2:** Clinical characteristics and stimulation parameters of the patients

Patient	Gender and Age at surgery (Years)	Duration (Years)	Etiology	Medication	Analgesic effect (VAS)	Stimulation parameters
1 ZYC	M/64	2	Ischemia (L-Thalamus)	Gabapentin, Carbamazepine, Oxycodone & n	9 - > 0	- [7] + [1 2] 160 μs; 40 Hz; 0.95 V
2 MWH	F/74	20	Hemorrhage (L-Thalamus)	Tramadol, Gabapentin	8 - > 4	- [1 3] + [2] 120 μs; 15 Hz; 0.5 V
3 SHP	M/60	7	Hemorrhage (R-Thalamus)	Baclofen, Tramadol, Gabapentin, Carbamazepine	7 - > 7	- [0 3] + [1 2] 60 μs; 15 Hz; 0.8 V

### Outcomes predictors

**Figure 3:**
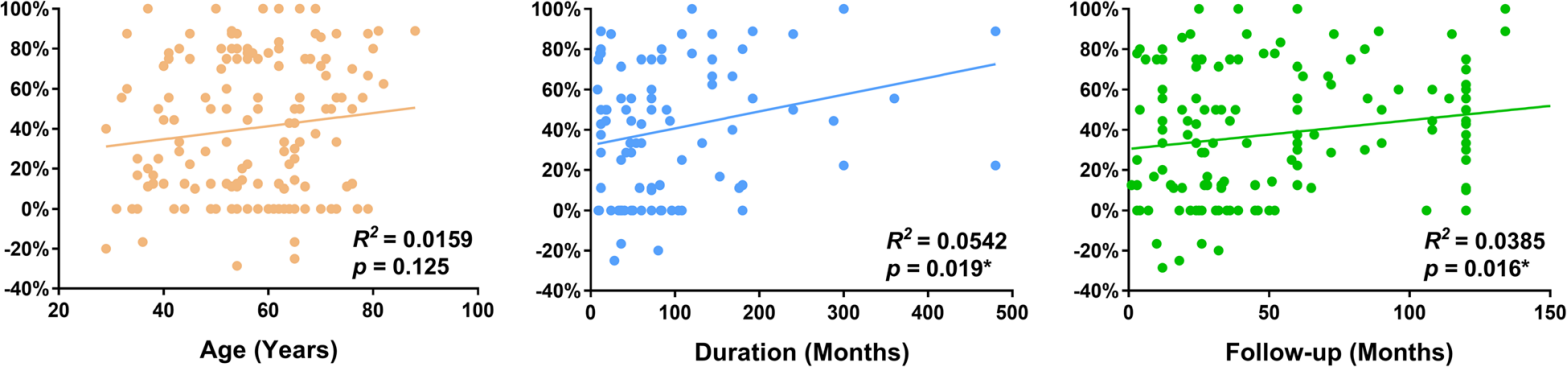
Scatterplot. Positive correlations between the percentage improvement of the VAS scores and predictive factors. *: Statistically significant

A linear regression model (Pearson’s correlation) was used to determine whether age, the disease duration and the follow-up time had relationships with the percentage of improvement in the VAS. According to the outcome, the coefficient value (r) was 0.126* with a *p* value of 0.125 in the age subgroup. No significant relationship was observed between age and post-operative improvement. However, in the duration subgroup, the coefficient value was 0.233 with a *p* value of 0.019, and in the follow-up time subgroup, the coefficient value was 0.196 with a *p* value of 0.016. A small positive correlation was found among the duration, follow-up time and postoperative improvement (Fig. 3).

## Discussion

### Achievement

As reasonable medical therapy was invalid for patients with intractable pain, MCS emerged as a new and promising treatment option. After systematically collecting and quantifiably investigating data from numbers of relevant articles, it turned out that MCS performed effective effect on refractory pain. The highest improvement rate was seen for post-radicular plexopathy (65.1%), although and MCS might aggravate the pain induced by a spinal cord injury. However, the small sample size was a limitation to draw these conclusions. The mean improvement of pain resulting from stroke was 35.2%, and there no significant differences were found among the stroke subgroups (total, lesion involving the thalamus and lesion outside the thalamus). The mechanism of post-stroke pain is widely accepted to be a complex process of network reorganization rather than a simple process of focal hyperexcitability or disinhibition [40]. MCS effectively attenuates pain by directly affecting activity in the somatosensory areas and thalamic nuclei and inhibiting spinal primary afferents and spinothalamic tract neurons [2]. Moreover, MCS takes part in modulation of a deeper and wider range of brain structures, such as the striatum, thalamic area, cerebellum, ventral posterolateral nucleus (VPL) and rostral agranular insular cortex (RAIC) [41, 42]. Patients with trigeminal neuropathic pain obtained 46.5% pain relief at the last follow-up. The explanations for the outcomes may be related to the facial area, which is one of the largest regions of the motor cortex [43]. The improvement rates of plexus avulsion and phantom pain were similar. This phenomenon may result from the same pathophysiological changes in both aetiologies: (i) altered activity in the neuromas after injury; ephaptic connections are formed after injuries in the periphery, which may result in increased afferent signaling, and increases in new connections may lower the threshold [44]; (ii) spinal segmental deafferentation [45]; and (iii) cortical reorganization of sensory fields [46, 47].

Meanwhile, both the duration of disease and the time of follow-up had small predicative value, while age had no correlation to post-operative VAS, which was rarely reported in previous articles. According to the existing literature, in response to operative repetitive transcranial magnetic stimulation (rTMS) or a pharmaceutical drug, the relatively intact cortico-spinal tract and the sensory system, experience pain relief in the first month, and motor weakness of the painful area are the predictors [48–51]. However, approximately about 30% of patients who did not show improvement by rTMS were improved by MCS [52], which might raise concerns for many clinicians regarding the cost-effectiveness ratio of this method due to the low negative predictive value. Regardless, preoperative rTMS is worth using. On one hand, the analgesic effects of preoperative rTMS may help clinicians predict a patient’s prognosis and increase the confidence of neurosurgeons performing MCS. On the other hand, the clinical effects of MCS are estimated not only by single predictors of the response to rTMS but also by a combination of other factors, including the different pain subtypes, duration, hyperpathia, and preoperative motor status. Therefore, preoperative rTMS is valuable. The explanation for the positive predictive value of rTMS is that the descending volleys elicited by epidural MCS are similar to those elicited by rTMS to produce analgesic effects [53, 54]. Direct wave (D-wave) and indirect waves (I-waves) are widely accepted as a mechanism of electrical stimulation of the brain cortex. The D-wave is the first valley resulting from direct stimulation of pyramidal tract axons and I-waves are the later volleys resulting from synaptic activation of the same pyramidal tract neurons. In addition, the morphology of pyramidal neurons in layer 5 of the motor cortex is crucial for generate of I-waves [54]. Generation of descending volleys depends on the electrode placement, montage, polarity and stimulus intensity. Bipolar MCS was confirmed to generate I3-waves capable of producing maximal pain relief, and the analgesic effects of MCS were related to activation of intracortical horizontal fibers or interneurons rather than the pyramidal tract [55].

The concrete mechanism of MCS remains elusive. Nevertheless, it was hypothesized that the potential mechanism might be correlated with several factors. Brasil-Neto and his colleagues considered that the corollary discharge reinforcement could deteriorate sensory feedback [56]. Increase of regional cerebral blood flow in the ipsilateral ventrolateral thalamus cingulate gyrus, orbitofrontal cortex, and brainstem may help to explain the mechanism of MCS [57, 58]. Besides, the activation of top-down controls related to the excitation of intracortical horizontal fibers [58] and perigenual cingulate and orbitofrontal areas may modulate the emotional appraisal of pain [59]. In Silva’s opinion, spinal anti-neuroinflammatory effect and the activation of the cannabinoid and opioid systems via descending inhibitory pathways [60]. Moreover, the basic researches also helped interpret the secret of MCS. The present treatment helped alleviate the level of glial acidic protein (GAP) in the anterior cingulate cortex (ACC) [61] and opioid and dopamine D1 receptors within the PAG participated the inhibitory effect of MCS [59, 62].

Considering the stimulation of anatomical region, it had been confirmed that stimulation of cortical regions adjacent to the primary motor cortex fail to produce similar analgesic effects [58], which was highlighted by Hosomi et,al, he agreed with the stimulation of central sulcus was more effectively than the precentral gyrus [63]. Also, subdural MCS provided similar therapeutic effect compared to the preferred extradural MCS in long-term follow-up studies [11, 18]. These together indicated that effective cortical region of MCS was very limited though the neuromodulatory process involve various brain region. The correct electrodes placement was important.

Obviously, MCS represents an effectively functional neurosurgery to attenuate the medically intractable pain symptom [11]. Modulation of MCS not only had analgesic effects but improvement in motor and sensory system [38]; (4) MCS could restore tactile and thermal sensory loss to some extent [64]. However, the advantages should not be highlighted excessively and the side effects need to focus on. According to a case report, patient with stroke underwent MCS experienced the supernumerary phantom arm [65]. Seizure related the abrupt increase in stimulation intensity, infection, postsurgical incisional pain, and transient cerebral edema were not uncommon but tolerable [11, 66]. Intensive reprogramming can recapture the benefit of MCS, with increased risk of seizures [67].

Concurrently, past decades witnessed significant breakthrough in this field. An increasing researches focused more on the preoperative rTMS as the auxiliary treatment possessed predictive value to access efficacy of MCS, especially the 20 Hz rTMS significantly ameliorated the pain [68, 69]. Ivanishvili et al. initially pointed out that the cyclization of MCS will improve pain relief as well as prolong battery life and delay the replacement [36]. Clinically, MCS could be considered as add-on therapy when patients with pain failure to response to the spinal cord stimulation (SCS). If patients were failed to the MCS, ziconotide intrathecal delivery represented the alternative therapy [70]. As opioid-receptor availability appears to be related to the efficacy of MCS, optogenetics-mediated MCS may help clinicians to select the candidates most likely to benefit from this procedure [71].

With the progression of science and technology, modern devices and medicine image post-processing technology contributed to the interpretation of the mechanism. The appearance of fMRI imaging helps precisely locate facial areas on the precentral gyrus and contributes to pain reduction [66]. According to the changes in cerebral flood flow (CBF) evaluated by positron emission tomography (PET), we could verify the participation of motor and premotor cortices, anterior cingulate and PAG to modulate chronic pain [29, 72]. Moreover, intraoperative motor evoked potentials (iMEPs) recording had predictive value and cathode generated best analgesic effect [73] and repetitive laser stimulation (RLS)-induced gamma-band oscillations (GBO) modulation could detect cortical pain process in unresponsive wakefulness syndrome [74].

### Limitations

Though we tried our best to retrieve all published articles, establish strict included criteria, choose the optimal statistical methods, the small sample and the poor design studies still influenced the reliability of our article. Well-designed studies, such as randomized controlled or randomized, double-blind, crossover studies, were expected to further verify the effectiveness of MCS. Also, we failed to eliminate the negative effect brought by the different stimulation parameters across centers. The individual stimulation parameters rendered the statistical work difficult.

### Applications and future work

Although the rapid development of the MCS, it was still unclear whether the therapy represents an advancing alternative treatment. Besides, the specified mechanism and limitations await further refinement. Lastly, the efficacy of MCS depends on the accurate electrode placement, individualized programming parameters, patient selections, and response to rTMS. Future work is needed to further illustrate the advancing treatment and potential mechanism, such as endogenous pain control, the interaction between motor and pain system [75], and the involved neural circuits [20]. New generation of stimulators and electrode design worth paying enough attention to. The optimal target should be evaluated preoperatively via the usage of advanced neurological functional and structural imaging. General, specialized, quantitative and objective evaluation criterion should be developed and adopted to accurately investigate the pain relief in the clinical trials. Even better would be to focus more on the quality of life and capacity for work of patients. Well-designed study can provide strong evidence to explain this question. Future researches about the comparisons and contrasts between MCS and other neuromodulatory techniques is expected. Also, based on the principle of patient first, in order to minimize patient trauma, invasive treatments could be replaced by revolutionary and promising non-invasive therapies, if there is no statistically significant different in cost-benefit aspect.

## Conclusion

MCS is conducive to the patients with refractory pain. The duration of disease and the time of follow-up can be regarded as predictive factor. Meanwhile, further studies are needed to reveal the mechanism of MCS and to reevaluate the cost-benefit aspect with better-designed clinical trials.

## Additional files

Additional file 1: Raw data. Brief description of the data: Raw data from each included article (XLSX 20 kb)

Additional file 2: **Figure S1.** Analysis of the aetiology and prognosis of pain. KS: Kolmogorov-Smirnov test; SW: Shapiro-Wilk test. (TIF 1671 kb)

## Abbreviations

ACC: Anterior cingulate cortex
CBF: Cerebral flood
CT: Computed tomography
fMRI: functional MRI
FU: Follow up
GAP: Glial acidic protein
GBO: Gamma-Band Oscillations
iMEPs: intraoperative Motor Evoked Potentials
MCS: Motor cortex stimulation
MRI: Magnetic resonance imaging
NA: Not available
PAG: Periaqueductal gray
PET: Positron emission tomography
RAIC: Rostral Agranular Insular Cortex
RLS: Repetitive laser stimulation
rTMS: repetitive Transcranial Magnetic Stimulation
SCS: Spinal cord stimulation
SD: Standard deviation
VAS: Visual analogue scale
VPL: Ventral posterolateral nucleus
