# Supplementary figures and images for: Motor cortex stimulation: a systematic literature-based analysis of effectiveness and case series experience
Source: BMC Neurol. 2019 Mar 29;19:48. doi: 10.1186/s12883-019-1273-y (PMC6440080; doi:10.1186/s12883-019-1273-y)

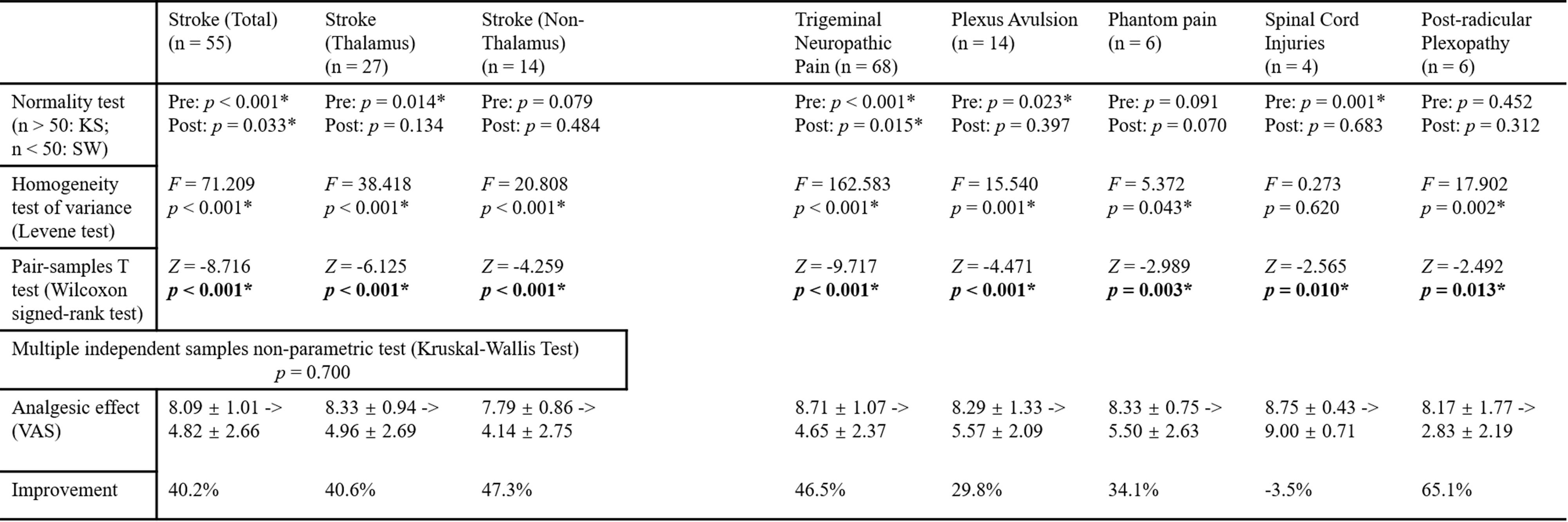

Supplement: Supplementary file 2 — Figure S1. Analysis of the aetiology and prognosis of pain. KS: Kolmogorov-Smirnov test; SW: Shapiro-Wilk test. (TIF 1671 kb) [file 12883_2019_1273_MOESM2_ESM.tif]
